# Parental rearing and psychopathology in mothers of adolescents with and without borderline personality symptoms

**DOI:** 10.1186/1753-2000-6-29

**Published:** 2012-08-27

**Authors:** H Marieke Schuppert, Casper J Albers, Ruud B Minderaa, Paul MG Emmelkamp, Maaike H Nauta

**Affiliations:** 1 Department of Psychiatry, University Medical Centre Groningen, Postbox 660, 9700 AR, Groningen, The Netherlands; 2 Department of Psychometrics and Statistical Methods, University of Groningen, Groningen, The Netherlands; 3 Department of Clinical Psychology, University of Amsterdam, Amsterdam, The Netherlands; 4 Department of Clinical Psychology, University of Groningen, Groningen, The Netherlands

**Keywords:** Borderline personality disorder, Adolescent, Rearing styles, Maternal psychopathology

## Abstract

**Background:**

A combination of multiple factors, including a strong genetic predisposition and environmental factors, are considered to contribute to the developmental pathways to borderline personality disorder (BPD). However, these factors have mostly been investigated retrospectively, and hardly in adolescents. The current study focuses on maternal factors in BPD features in adolescence.

**Methods:**

Actual parenting was investigated in a group of referred adolescents with BPD features (N = 101) and a healthy control group (N = 44). Self-reports of perceived concurrent parenting were completed by the adolescents. Questionnaires on parental psychopathology (both Axis I and Axis II disorders) were completed by their mothers.

**Results:**

Adolescents reported significantly less emotional warmth, more rejection and more overprotection from their mothers in the BPD-group than in the control group. Mothers in the BPD group reported significantly more parenting stress compared to mothers in the control group. Also, these mothers showed significantly more general psychopathology and clusters C personality traits than mothers in the control group. Contrary to expectations, mothers of adolescents with BPD features reported the same level of cluster B personality traits, compared to mothers in the control group. Hierarchical logistic regression revealed that parental rearing styles (less emotional warmth, and more overprotection) and general psychopathology of the mother were the strongest factors differentiating between controls and adolescents with BPD symptoms.

**Conclusions:**

Adolescents with BPD features experience less emotional warmth and more overprotection from their mothers, while the mothers themselves report more symptoms of anxiety and depression. Addition of family interventions to treatment programs for adolescents might increase the effectiveness of such early interventions, and prevent the adverse outcome that is often seen in adult BPD patients.

## Background

Borderline personality disorder (BPD) in adolescence places a significant burden on patients and their families and often has negative long-term effects on a broad range of domains, such as recurrent Axis I pathology, poor general functioning, and problems in relationships and self-care [1,2].

A combination of strong genetic predisposition and environmental factors is considered as a model for the development of BPD [3-5]. Several studies have found an increased risk of BPD in families, especially in first-degree relatives [6-8]. Next to genetic factors, several psychosocial factors have been identified as risk factors for the development of BPD. For instance, growing up in a dysfunctional family, parental rearing styles, and early childhood adversities have all been found to be related to the development of BPD traits [9]. The main theories on the relationship between family factors and the development of BPD, are psychoanalytic. It has been suggested that BPD has its cause in mothers that did not allow their child to separate, i.e. were overprotective [10]. A strong association between BPD and insecure and disorganized forms of attachment has been found, [11] which is in line with theories that regard interpersonal instability as one of the core symptoms of BPD. In the last decades, in addition to attachment theory there is an increasing interest in the interaction between parenting, genetically influenced temperamental factors, childhood adversities, and parental psychopathology [9,12]. Though the scientific support is still weak, several theories suggest a causal relationship between separation and/or attachment problems and the development of BPD [11,13,14]. Next to attachment theory, cognitive theories have also sought for a developmental model of BPD [15-17]. For example, Linehan [15] poses that BPD results from growing up in an invalidating environment toward the expression of emotions. Another theory, developed by Young, [16] presumes that BPD is characterised by pervasive patterns of thinking, behaving, and feeling. These maladaptive patterns develop when basic childhood needs are met inadequately.

It is conceivable that problems in early childhood are even a larger challenge for parents that face psychopathological problems themselves. The current paper investigates the role of maternal rearing as well as maternal psychopathology in relation to BPD features (as described in DSM-IV) in youth with current BPD features, and healthy controls.

Parental rearing factors are widely presumed to be of substantial influence in the development of BPD. Findings from these studies are robust and impressive. For instance, 92% of the adult BPD patients reported a history of emotional neglect (assessed with seven items in a semi-structured interview) in a study with 358 BPD patients and 109 patients with another PD [18]. Perceived lack of maternal care, as retrospectively assessed by attachment and parental bonding instruments, was found to be associated with BPD traits in a community sample of 18-year-old students with BPD features (N = 393), selected from a group of 5000 students [19]. More recently, negative parenting styles (rejection and overprotection), as well as conflictive parenting, were found to be associated with the occurrence of personality disorder (PD) in general, but this was not specifically investigated for borderline PD [20]. This study concerned a community sample of 181 students with personality disorder (as determined with the International Personality Disorder Examination, IPDE) and 2605 controls. In conclusion, adults with BPD consistently report on a history of less parental warmth, more rejection and hostility, and more overprotection in their childhood.

However, all aforementioned studies are retrospective, and no assessments were made in parents. Though retrospective studies are valuable, it is well-known that they are prone to recall bias, [21] and interpretation of these studies needs caution. Only few studies investigated actual parenting behaviour in relation to BPD features in adolescents, all in community samples. Only one study reported on parental overprotection: the Children in the Community (CIC) study (N = 776) reported that maternal overinvolvement had no direct impact on a persistence or an emergence of BPD 2.5 years later. However, the combination of maternal overinvolvement with maternal inconsistency was a predictor of BPD [22]. Assessments were made both in mothers and their offspring. In another paper on the CIC study, low parental affection and aversive parenting were both associated with an elevated risk for BPD in the offspring [23]. Maternal hostility was also associated with BPD features in adults in a community sample of mothers (N = 162) with low income [24]. So, the studies within childhood are scarce, all community-based, and the evidence for the role of parental rearing is much less clear and less strong than retrospective research suggests.

The present study compares current maternal parenting behaviour in a clinical sample of referred adolescents (14–19 years) with BPD features (N = 101) to a healthy control group (N = 44). Data from the adolescents (on concurrent maternal rearing), as well as from their biological mothers (on maternal psychopathology), were collected. Based on previous research on the development of BPD, we hypothesised adolescents with BPD features to report less emotional warmth, more rejection, and more overprotection, as compared to healthy controls.

Parental rearing may be interwoven with parental psychopathology. Indeed, a recent overview focusing on parenting behavior of mothers with BPD concludes that several factors play a part in the poor psychosocial functioning that has been found in their children [25]. Among these factors are insensitive communication (critical, intrusive, and frightening), role confusion (i.e. addressing the child as a friend or parent), and increased risk of abuse. In line with these findings, stronger associations between negative parenting styles and personality disorder symptoms were found in students that grew up with a parent with PD than in students that grew up with a parent without PD, [20] but this was not investigated specifically for borderline PD.

Parental psychopathology may also have an impact on the development of BPD on its own, even though it may not be a specific relation, and the evidence seems mixed: maternal BPD increases the risk for a range of emotional and behavioral problems, including BPD [25]. Contrary to expectations, no elevated risk for PDs in offspring of parents with psychiatric disorders was found in the CIC study, though this was extensively investigated using several standardised interviews with mothers and offspring [23,26]. White et al. [27] reviewed 59 studies to examine the literature on psychopathology in BPD patients and their relatives. They found no link between BPD and schizophrenia, an ambiguous link between BPD and major depressive disorder, and a possible familial aggregation of impulse spectrum disorders (including BPD) and BPD. Parental psychopathology is also associated with an increased risk for suicidal behavior in the offspring. A large study (N = 55299) conducted in 21 countries all over the world, revealed that parental generalized anxiety disorder and depression were predictors for suicidal plans, and that parental antisocial behavior and anxiety disorders were predictors for suicide attempts [28]. In the current study, we explored differences in general psychopathology and personality symptoms between mothers of referred adolescents with BPD features and healthy controls. In view of the transgenerational transmission of BPD, [25] we expect mothers of BPD adolescents to report more cluster B symptoms and more general psychopathology compared to mothers in the healthy control group.

The current study is unique in that it investigates all of the aforementioned factors in a clinical sample of adolescents with BPD features, and a healthy control group, and their mothers. Concurrent parental rearing styles (perceived overprotection, rejection, emotional warmth), and psychopathology in mothers (mother-reported general psychopathology and personality traits) were entered in a hierarchical logistical regression model, to examine which factors account for differences between adolescents with BPD features and healthy controls.

## Methods

### Participants

Participants were adolescents aged 14 to 19 and their mothers. The adolescents of the clinical sample (N = 101) were referred to the Emotion Regulation Training, a group training for adolescents with BPD features (ERT) [29]. Inclusion criteria were: age 14–19, IQ ≥ 80 (according to school results), at least two borderline symptoms as assessed by SCID-II [30]. The mean number of BPD criteria was 6.02 (SD 1.99) out of nine; 75.2% fulfilled full criteria for a BPD diagnosis. Adolescents with psychotic disorders, conduct disorder, or serious misuse of drugs or alcohol were excluded from the study. The corresponding sections of the Kiddie-Schedule for Affective Disorders and Schizophrenia for School-Age Children – Present and Lifetime version (K-SADS-PL) [31] were used to examine these exclusion criteria.

Healthy controls and their mothers (N = 44) were recruited through letters, posters, and mouth-to-mouth at secondary schools. They had never been referred or treated for mental health problems.

The ethical committee of the Department of Psychology Groningen approved of this study. Written informed consent of all participants and mothers was obtained after extensive information about the study. Demographic variables of the participants are shown in Table 1.

**Table 1:** Demographics

	**Clinical group (N = 101)**^**#**^	**Control group (N = 44)**	**Comparison (Fisher’s exact test)**
Age (SD)	16.32 (1.15)	15.93 (1.20)	p = .72
Women	97 (96.0%)	37 (84.1%)	p = .02
Parents divorced	48 (50.0%)	8 (18.2%)	p < .000
Contact with justice	29 (29.9%)	6 (13.6%)	p = .06
Non-Dutch parent	16 (16.3%)	4 (9.1%)	p = .31

^#^ Due to missing data n varies from 96 – 101.

#### Measures

The *Structured Clinical Interview for DSM-IV Personality Disorders – borderline personality disorder section*[30] was used to assess borderline pathology. This instrument has been developed for adults, but is frequently used in adolescents as well [32].

*Kiddie Schedule for Affective Disorders and Schizophrenia for School-Age Children – Present and Lifetime version* (K-SADS-PL) [31] is a semi-structured interview based on DSM-IV. We used the modules disruptive behavior disorders and psychotic disorders to obtain information on exclusion criteria.

Perceived parenting was measured by *EMBU-*C [33]*.* EMBU is a Swedish acronym for ‘my memories of upbringing’. EMBU-C is a self-report for youth and adaptation of the original retrospective self-report. It is a frequently used and well-evaluated instrument [33-35]. Three factors were included in the current study: Emotional Warmth (19 items), Rejection (17 items) and Overprotection (11 items).

The *Symptoms Checklist-90-R* (*SCL-90-R*) [36] is a frequently used self-report questionnaire consisting of 90 items, that assesses general psychopathological complaints. In the current study, the list was completed by the mothers. Validity and reliability of the SCL-90-R have shown to be good [37,38].

The *Personality Disorders Questionnaire 4+ (PDQ-4+*) [39] is a self-report questionnaire assessing personality disorders (PDs) as described in DSM-IV. It consists of 99 true/false items. We used the Dutch version by Akkerhuis et al. [40]. The PDQ was added to assess personality traits in mothers; we used the sum scores of cluster A, B, and C personality traits respectively.

#### Data analysis

SPSS-19 was used to analyse all data, with 5% significance levels. Second ratings on a random sample of taped interviews (both groups 10%) were made by the first author. Intraclass correlation coefficients (ICCs) were calculated to assess interrater reliability. Since the data was not normally distributed, nonparametric Mann–Whitney tests were conducted to compare differences between groups. Logistic regression analyses were performed to examine to what extent maternal rearing styles and psychopathology in mothers contribute to severity of borderline symptoms. All variables on maternal rearing styles and maternal psychopathology were entered in the model, followed by a stepwise removal of non-significant variables. The final model consisted of variables that all have a unique and significant correlation with BPD features.

## Results

The Intraclass Correlation Coefficients (ICCs) for the nine symptoms of the SCID-II-BPD section were all excellent, ranging from 0.89 to 0.97.

Table 2 presents the differences in maternal rearing style as perceived by adolescents, between adolescents with BPD features and healthy controls. In the borderline group, adolescents reported significantly less emotional warmth, more overprotection and more rejection as maternal rearing styles. Contrary to expectations, there was no significant difference between groups on cluster B personality traits in mothers, but mothers in the clinical group reported significantly more cluster C personality traits compared to mothers in the control group. No significant differences were found on cluster A personality traits. However, differences in general psychopathology were highly significant between mothers of adolescents with BPD features and mothers of healthy controls.

**Table 2:** Differences between groups (Means (and SD))

	**Clinical group (N = 96-101)**	**Control group (N = 44)**	**Comparison (U)**	**Effect size (Cohen’s d)**
**Mothers**				
PDQ cluster A	3.80 (3.76)	2.91 (3.73)	1746.50	.24
cluster B	3.24 (2.93)	3.04 (2.98)	1989.50	.07
cluster C	5.15 (3.27)	3.92 (3.45)	1539.50^**^	.37
SCL-90Total score	127.87 (33.34)	110.80 (21.68)	1402.50^**^	.61
**Adolescents**				
EMBU -EW	55.29 (12.16)	61.98 (9.54)	3964.50^***^	-.61
-R	27.61 (7.04)	22.55 (4.00)	1132.50^***^	.88
-O	24.54 (5.31)	21.08 (5.25)	1381.00^***^	.66

^**^p < .01; ^***^p < .000.

Mann–Whitney test between groups. *EMBU* = parental rearing style child version; *EW* = emotional warmth; R = rejection; *O* = overprotection; *AC* = affectionless control; *SCL-90* = symptoms checklist 90; *PDQ* cluster *A/B/C* = personality disorders questionnaire cluster A/B/C (corresponds with DSM-IV).

Due to missing values n varies from 140–145.

Table 3 presents the results of the hierarchical logistic regression analyses. The non-significant variables were removed in the following order: cluster A personality traits in mothers, rejection (rearing style), cluster C, and lastly cluster B personality traits in mothers. The three remaining variables (general psychopathology of the mother, and rearing styles: emotional warmth and overprotection) were all significantly associated with borderline symptoms in the adolescent. Nagelkerke R [2] was .30.

**Table 3:** Logistic regression of parental rearing styles and psychopathology in mothers

	**Wald**	***p*value**	**OR**
Constant	.27	.602	.41
Emotional warmth	10.40	.001	.93
Overprotection	10.50	.001	1.16
General psychopathology (SCL-90)	5.06	.024	1.02

Dependent variable: group membership (adolescents with BPD symptoms versus controls); SCL-90 = symptoms checklist 90; OR = unadjusted odds ratio.

Nagelkerke R2 = .30.

The variables rejection (parental rearing style), and cluster A, B, and C (personality disorder symptoms) were excluded from the model due to non-significance.

## Discussion

Borderline personality disorder is a frequently studied condition that has its roots in childhood and adolescence, and is caused by multiple factors [3,5-12]. Though it is widely accepted that some of these factors lay within family circumstances and parental rearing, this has mostly been evaluated retrospectively in community samples rather than clinical samples. In this study we examined perceived maternal rearing styles, and maternal psychopathology in a group of 101 adolescents with BPD features, 44 healthy controls, and their mothers. One of the strengths of our study is that the adolescents reported on perceived concurrent rearing by their parents, which makes the assessment less affected by recall bias. Also, this is the first study to report on referred adolescents with BPD features. Furthermore, to our knowledge, there is a paucity of research of parental psychopathology, both on Axis I and Axis II problems [20].

The main results of our study are as follows: (1) Adolescents with current elevated levels of BPD features report higher levels of maternal rejection, overprotection, and lower emotional warmth. (2) Mothers of adolescents with BPD features report more general psychopathology and cluster C personality symptoms, but no more cluster A and cluster B symptoms. (3) Three variables were the strongest predictors of BPD features in adolescents, namely the maternal rearing styles less emotional warmth and more overprotection, and more general psychopathology in mothers.

Contrary to expectations, we found no elevated levels of maternal cluster B personality traits in mothers of adolescents with BPD features. However, we did find higher levels of maternal cluster C traits in this group. Our findings differ from the study of Gunderson et al. [6], who found a 3- to 4- fold increased level of BPD in first-degree probands of BPD patients. Cheng et al. [20] found an increased risk for PD in students that were raised by a parent with personality pathology, but differences in (clusters of) parental or student PDs were not reported in their study. The relatively low level of cluster B personality traits in mothers of the clinical group is remarkable and might be due to methodological weaknesses in our study, such as the small sample size in the control group and the use of a self-report questionnaire for personality traits in mothers. However, our self-report measure on maternal BPD (PDQ-4) usually leads to higher rather than lower estimates of BPD. Next to the transgenerational transmission model, [25] another factor in the pathway to BPD is assumed to be dysfunctional parenting. Several studies have found ample evidence for this relationship [20,23,25,26,28]. This may be an explanation for the increased levels of cluster C traits in our sample: mothers with increased levels of cluster C (anxious, fearful) personality traits, may raise their children with more overprotection, and thus increase the risk of BPD in their offspring. Even more, in combination with general psychopathology (like anxious/depressive symptoms), those mothers may be unstable and unpredictable, and thus arouse instability in children that are already vulnerable for the development of BPD features.

Most (psychodynamic) theories on the development of BPD suggest that inappropriate parenting, like a lack of emotional warmth, high levels of parental criticism/regression, or overprotection, increase the risk of BPD symptoms. However, adolescents with BPD features may provoke these parenting behaviors, by their impulsive, instable, and dangerous behavior. These two causal directions may even reinforce each other [41]. In addition, also other theories, including learning theories, have described the putative mechanisms involved in the development of BPD, and have included both parenting factors and offspring factors [13-17].

In our study, the maternal rearing styles emotional warmth and overprotection, together with increased general psychopathology in mothers, were associated with BPD features in adolescents. The model was able to classify 70% of the adolescents correctly (i.e. being assigned to the clinical or the control group) by using these three variables. Our findings are in line with the CIC study, [23] who found an association between aversive parental behavior and low parental affection, and BPD. However, they found no direct association between parental psychiatric disorders and increased risk for offspring PD. As in our study, Cheng et al. [20] found negative and conflicting parenting styles to be associated with the occurrence of personality disorder in general.

We found higher levels of emotional warmth in the control sample then in the clinical sample. Both parent and adolescent personality factors have been found to be relevant for influencing parenting behavior [42]. High levels of extraversion and agreeableness in adolescents, and high levels of agreeableness in parents, results in better, positive parenting. It has been suggested that emotional stable parents are less anxious, and are therefore better able to handle problematic behavior in their adolescent children [42]. Further exploration of moderators in the pathway to BPD is necessary, in order to develop interventions that aim at specific components of the disorder.

The contribution of parenting styles and maternal psychopathology is not unique to BPD features in youngsters [43]. The same factors have been found to be associated with anxiety disorders [44] and depression [45]. For example, overprotective parenting has also been found in a recent study with children with anxiety disorders (N = 190, age 7–13 years) [46]. It is yet unclear what specific pathway leads to specific psychopathology.

There are, of course, some limitations. We used a cross-sectional design, so no causal interferences can be made. Though the sample size of the clinical group is large, the sample size of the control group is moderate. Further, our sample consisted almost only of girls, so generalization to a mixed population needs caution. On the other hand, this seems to reflect the general gender distribution among referred adolescents with BPD. Also, we did not report on severity of borderline symptoms. We used the self-report PDQ-4 to assess traits of personality disorders in mothers, an instrument that is known as sensitive, but also as non- specific. Moreover, we did not use a formal instrument to diagnose Axis I and Axis II disorders, not in mothers nor in adolescents (except for BPD symptoms in adolescents). It cannot be ruled out the results would have been different if we used a formal diagnostic interviews, e.g. the SCID-I [47] and SCID-II [48]. Also, data on traumatic experiences is lacking. Another limitation is that we selected adolescents that already reported BPD symptoms. Their scores on the parental rearing scales may be affected by a response or attribution bias.

Another limitation is that we only reported on maternal rearing, leaving fathers out of consideration. Studies on BPD that included fathers in the assessment are scarce. The CIC- study reported on paternal rearing and paternal psychopathology, but the information was obtained through maternal interviews [23]. Gureje et al. [28] did obtain information from both parents, but they did not report separately for fathers and mothers. However, it is likely that the role of fathers differs from the role of mothers. For example, it has been found that fathers influence the behavior of socially anxious children more than mothers [49]. Bögels and Phares [50] propose a model for the role of paternal rearing in the development of anxiety disorders. They underline the importance of the role of fathers in the transition to the outer world, including encouragement of independence and appropriate risk-taking. The same may hold for adolescents with BPD and their fathers. Exploring the unique ways in which both fathers and mothers are involved in the etiology of BPD, is an interesting topic for future research.

## Conclusions

Our study aims to contribute to solution of the complex puzzle of the pathogenesis of BPD. Adolescents with BPD features indeed report to be raised by less emotional warm, more overprotective, and more rejective mothers than healthy controls. Their mothers are more anxious / fearful than controls. Up to now, only few age-specific interventions for BPD symptoms in adolescents have been developed. Even less interventions have been evaluated, [51] and the results of these interventions are disappointing [1,52,53]. It is particularly notable that most interventions pay little attention to parents or caretakers. However, systemic interventions might focus on parenting skills and help parents to show more warmth to their adolescents and to encourage the adolescent to become independent in a responsible way. Improvement of maternal anxiety and mood related symptoms might not only benefit themselves, but also their children. Mutual understanding of mechanisms that contribute to interpersonal difficulties in families may help both adolescents and their parents to reduce obstructions in their relationship. Early interventions, not only in young individuals, but also in families, might prevent the adverse outcome that is often seen in adult BPD patients.

## Competing interests

The first author has been financially supported by a ZonMW grant, the Netherlands organisation for health research and development, number 10-000-2030, to the first author.

## Authors’ contributions

HMS was responsible for the coordination of the study and the manuscript. CJA participated in the design of the study and the statistical analyses. RBM contributed to the design of the study and was involved in revising the manuscript. PMGE participated in the design of the study, the interpretation of the data and revising the manuscript. MHN made substantial contributions to the design of the study, the analysis and interpretation of the data, and drafting and revising the manuscript. All authors read and approved the final manuscript.

## References

[B1] Chanen AM Jackson HJ McCutcheon LK Jovev M Dudgeon P Yuen HP Germano D Nistico H McDougall E Weinstein C Clarkson V McGorry PD Early intervention for adolescents with borderline personality disorder using cognitive analytic therapy: randomised controlled trial Br J Psychiatry 2008 193 477 484 10.1192/bjp.bp.107.048934 19043151

[B2] Crawford TN Cohen P First MB Skodol AE Johnson JG Kasen S Comorbid Axis I and Axis II disorders in early adolescence: prognosis 20 years later Arch Gen Psychiatry 2008 65 641 648 10.1001/archpsyc.65.6.641 18519822

[B3] Chanen AM Kaess M Developmental pathways to borderline personality disorder Curr Psychiatry Rep 2012 14 45 53 10.1007/s11920-011-0242-y 22009682

[B4] Crowell SE Beauchaine TP Linehan MM A biosocial developmental model of borderline personality disorder: elaborating and extending Linehan’s theory Psychol Bull 2009 135 495 510 19379027 10.1037/a0015616 PMC2696274

[B5] Distel MA Middeldorp CM Trull TJ Derom CA Willemsen G Boomsma DI Life events and borderline personality features: the influence of gene-environment interaction and gene-environment correlation Psychol Med 2011 41 849 860 10.1017/S0033291710001297 20594379

[B6] Gunderson JG Zanarini MC Choi-Kain LW Mitchell KS Jang KL Hudson JI Family study of borderline personality disorder and its sectors of psychopathology Arch Gen Psychiatry 2011 68 753 762 10.1001/archgenpsychiatry.2011.65 21727257 PMC3150490

[B7] Johnson JG Brent DA Connolly J Bridge J Matta J Constantine D Rather C White T Familial aggregation of adolescent personality disorders J Am Acad Child Adolesc Psychiatry 1995 34 798 804 10.1097/00004583-199506000-00021 7608054

[B8] Skodol AE Shea MT Yen S White CN Gunderson JG Personality disorders and mood disorders: perspectiveson diagnosis and classification from studies of longitudinal course and familial aggravations J Pers Disord 2010 24 83 108 10.1521/pedi.2010.24.1.83 20205500 PMC6540749

[B9] Paris J Personality disorders over time. Precursors, course, and outcome 2003 Washington, DC: American Psychiatric Publishing, Inc 10.1521/pedi.17.6.479.25360 14744074

[B10] Materson J Rinsley D The borderline syndrome: role of the mother in the genesis and psychic structure of the borderline personality Int J Psychoanal 1975 56 163 177 1158575

[B11] Agrawal HR Gunderson J Holmes BM Lyons-Ruth K Attachments studies with borderline patients: a review Harv Rev Psychiatry 2004 12 94 104 10.1080/10673220490447218 15204804 PMC1857277

[B12] Paris J Treatment of borderline personality disorder. A guide to evidence-based practice 2008 New York, London: The Guilford Press

[B13] Fonagy P Bateman A The development of borderline personality disorder – a mentalizing model J Pers Disord 2008 22 4 21 10.1521/pedi.2008.22.1.4 18312120

[B14] Kernberg OF Yeomans FE Clarking JF Levy KN transference focused psychotherapy: overview and update Int J Psychoanal 2008 89 601 620 10.1111/j.1745-8315.2008.00046.x 18558958

[B15] Linehan MM Cognitive-behavioral treatment of borderline personality disorder 1993 New York: The Guilford Press

[B16] Kellogg SH Young JE Schema therapy for borderline personality disorder J Clin Psychol 2006 62 445 458 10.1002/jclp.20240 16470629

[B17] Ryle A The contribution of cognitive analytic therapy to the treatment of borderline personality disorder J Pers Disord 2004 18 3 35 10.1521/pedi.18.1.3.32773 15061342

[B18] Zanarini MC Williams AA Lewis RE Bradford Reich R Vera SC Marino MF Levin A Yong L Frankenburg FR Reported pathological childhood experiences associated with the development of borderline personality disorder Am J Psychiatry 1997 154 1101 1106 9247396 10.1176/ajp.154.8.1101

[B19] Nickell AD Waudby CJ Trull TJ Attachment, parental bonding and borderline personality disorder features in young adults J Personal Disord 2002 16 148 159 10.1521/pedi.16.2.148.22544 12004491

[B20] Cheng HG Huang Y Liu Z Liu B Associations linking parenting styles and offspring personality disorder are moderated by parental personality disorder, evidence from China Psychiatry Res 2011 189 105 109 10.1016/j.psychres.2010.12.006 21195487

[B21] Maughan B Rutter M Retrospective reporting of childhood adversity: assessing long-term recall J Personal Disord 1997 11 19 33 10.1521/pedi.1997.11.1.19 9113820

[B22] Bezirganian A Cohen P Brook JS The impact of mother-child interaction on the development of borderline personality disorder Am J Psychiatry 1993 150 1836 1842 8238639 10.1176/ajp.150.12.1836

[B23] Johnson JG Cohen P Chen H Kasen S Brook JS Parenting behaviours associated with risk for offspring personality disorder during adulthood Arch Gen Psychiatry 2006 63 579 587 10.1001/archpsyc.63.5.579 16651515

[B24] Carlson EA Egeland B Sroufe LA A prospective investigation of the development of borderline personality symptoms Dev Psychopath 2009 21 1311 1334 10.1017/S0954579409990174 PMC11034778 19825270

[B25] Stepp SD Whalen DJ Pilkonis PA Hipwell AE Levine MD Children of mothers with borderline personality disorder: identifying parenting behaviors as potential targets for intervention Personal Disord: Theory, Research, Treatment 2011 3 1 76 91 10.1037/a0023081 PMC3268672 22299065

[B26] Johnson JG Liu L Cohen P Parenting behaviours associated with the development of adaptive and maladaptive offspring personality traits Can J Psychiatry 2011 56 447 456 21878155 10.1177/070674371105600802

[B27] White CN Gunderson JG Zanarini MC Hudson JI Family studies of borderline personality disorder: a review Harv Rev Psychiatry 2003 11 8 19 10.1080/10673220303937 12866737

[B28] Gureje O Oladeji B Hwang I Chiu WT Kessler RC Sampson NA Alonso J Andrade LH Beautrais A Borges G Bromet E Bruffaerts R de Girolamo G de Graaf R Gal G He Y Hu C Iwata N Karam EG Kovess-Masféty V Matschinger H Moldovan MV Posada-Villa J Sagar R Scocco P Seedat S Tomov T Nock MK Parental psychopathology and the risk of suicidal behavior in their offspring: results from the World Mental Health surveys Mol Psychiatry 2011 16 1221 1233 10.1038/mp.2010.111 21079606 PMC3142278

[B29] TM Gv Ringrose HJ Schuppert HM Wiersema HM Emotieregulatietraining (ERT), een programma voor adolescenten met emotieregulatie problemen 2009 Amsterdam: Boom

[B30] Weertman A Arntz A Kerkhofs MLM: Gestructureerd klinisch interview voor DSM-IV persoonlijkheidsstoornissen (SCID-II) 2000 Lisse: Swets & Zeitlinger

[B31] Kaufman J Birmaher B Brent D Rao U Flynn C Moreci P Williamson D Ryan N Schedule for affective disorders and schizophrenia for school-aged children – present and life-time version (K-SADS-PL): initial reliability and validity data J Am Acad Child Adolesc Psychiatry 1997 36 980 988 10.1097/00004583-199707000-00021 9204677

[B32] Chanen AM Jovev M Djaja D McDougall E Yuen HP Rawlings D Jackson HJ Screening for borderline personality disorder in outpatient youth J Personal Disord 2008 22 353 364 10.1521/pedi.2008.22.4.353 18684049

[B33] Markus MT Lindhout IE Boer F Hoogendijk THG Arrindell WA Factors of perceived parental rearing styles: the EMBU-C examined in a sample of Dutch primary school children Personal Ind Diff 2003 34 503 519 10.1016/S0191-8869(02)00090-9

[B34] Aluja A Del Barrio V Garcia LF Do parents and adolescents differ in their perceptions of rearing styles? Analysis of the EMBU versions for parents and adolescents Scand J Psychol 2006 47 103 108 10.1111/j.1467-9450.2006.00497.x 16542352

[B35] Oldehinkel AJ Veenstra R Ormel J de Winter AF Verhulst FC Temperament, parenting, and depressive symptoms in a population sample of preadolescents J Child Psychol Psychiatry 2006 47 684 695 10.1111/j.1469-7610.2005.01535.x 16790003

[B36] Derogatis LR Lipman RS Covi L SCL-90: an outpatient psychiatric rating scale - preliminary report Psychopharmacol Bull 1973 9 1 13 28 4682398

[B37] Arrindell WA Barelds DPH Janssen ICM Buwalda FM van der Ende J Invariance of SCL-90-R dimensions of symptom distress in patients with per partum pelvic pain (PPPP) syndrome Br J Clin Psychol 2006 45 377 391 10.1348/014466505X68924 17147103

[B38] Olsen LR Mortensen EL Bech P The SCL-90 and SCL-90-R validated by item response models in a Danish community sample Acta Psychiatr Scand 2004 110 225 229 10.1111/j.1600-0447.2004.00399.x 15283743

[B39] Hyler SE Personality Diagnostic Questionnaire-4+ 1994 New York: New York State Institute

[B40] Akkerhuis GW Kupka RW van Groenestein MAC Nolen WA PDQ-4+ vragenlijst voor persoonlijkheidskenmerken: experimentele versie 1996 Lisse: Swets & Zeitlinger

[B41] Gunderson JG Lyons-Ruth K BPD’s interpersonal hypersensitivity phenotype: a gene-environment-developmental model J Pers Disord 2008 22 22 41 10.1521/pedi.2008.22.1.22 18312121 PMC2596628

[B42] Haan AD Dekovic M Prinzie P Longitudinal impact of parental and adolescent personality on parenting J Personal Soc Psychol 2012 102 189 199 10.1037/a0025254 21875227

[B43] Weich S Patterson J Shaw R Stewart-Brown S Family relationships in childhood and common psychiatric disorders in later life: systematic review of prospective studies Br J Psychiatry 2009 194 392 398 10.1192/bjp.bp.107.042515 19407266

[B44] Lieb R Wittchen H-U Höfler M Fuetsch M Stein MB Merikangas KR Parental psychopathology, parenting styles, and the risk of social phobia in offspring Arch Gen Psychiatry 2000 57 859 866 10.1001/archpsyc.57.9.859 10986549

[B45] Weissman MM Wickramaratna P Nomura Y Warner V Verdeli H Pilowsky DJ Grillon C Bruder G Families at high and low risk for depression: a 3-generation study Arch Gen Psychiatry 2005 62 29 36 10.1001/archpsyc.62.1.29 15630070

[B46] Gere MK Villabø MA Torgersen S Kendall PC Overprotective parenting and child anxiety: the role of co-occuring child behavior problems Anxiety Disord 2012 26 642 649 10.1016/j.janxdis.2012.04.003 22659077

[B47] First MB Spitzer RL Gibbon M Williams JBW Structured Clinical Interview for DSM-IV (SCID-I) 1997 Washington: American Psychiatric Press

[B48] First MB Gibbon M Spitzer RL Williams JBW Benjamin LS User’s guide for the structured clinical interview for DSM-IV Axis II personality disorders (SCID-II) 1997 Washington DC: American Psychiatric Press

[B49] Bögels S Stevens J Majdandžić M Parenting and social anxiety: father’s versus mother’s influence on their children’s anxiety in ambiguous social situations J Child Psychol Psychiatry 2011 52 599 606 10.1111/j.1469-7610.2010.02345.x 21155774

[B50] Bögels S Phares V Fathers’ role in the etiology, prevention and treatment of child anxiety: A review and a new model Clin Psychol Rev 2008 28 539 558 10.1016/j.cpr.2007.07.011 17854963

[B51] Ougrin D Tranah T Leigh E Taylor L Rosenbaum Asarnow J Practitioner review: self-harm in adolescents J Child Psychol Psychiatry 2012 53 337 350 10.1111/j.1469-7610.2012.02525.x 22329807

[B52] Schuppert HM Giesen-Bloo J van Gemert TG Wiersema H Minderaa RB Emmelkamp PMG Nauta MH Effectiveness of an emotion regulation group training for adolescents – a randomized controlled pilot study Clin Psychol Psychother 2009 16 467 478 10.1002/cpp.637 19630069

[B53] Schuppert HM Timmerman ME Bloo J van Gemert TG Wiersema HM Minderaa RB Emmelkamp PMG Nauta MH Emotion regulation training for adolescents with borderline personality disorder traits: a randomized controlled trial Submitted 10.1016/j.jaac.2012.09.002 23200288

